# *Lactobacillus delbrueckii* subsp. *bulgaricus* strain TCI904 reduces body weight gain, modulates immune response, improves metabolism and anxiety in high fat diet-induced obese mice

**DOI:** 10.1007/s13205-022-03356-3

**Published:** 2022-11-04

**Authors:** Yung-Kai Lin, Yung-Hsiang Lin, Chi-Fu Chiang, Tsung-Ming Yeh, Wen-Ling Shih

**Affiliations:** 1 Institute of Food Safety and Risk Management, Department of Food Science, National Taiwan Ocean University, Keelung, Taiwan; 2 Research & Design Center, TCI Co., Ltd., Taipei, Taiwan; 3 Department of Biological Science and Technology, National Pingtung University of Science and Technology, 1, Shuefu Rd., Neipu, Pingtung 91201 Taiwan; 4 General Research Service Center, National Pingtung University of Science and Technology, Neipu, Pingtung Taiwan

**Keywords:** *Lactobacillus delbrueckii* subsp. *bulgaricus*, Insulin resistance, Atherogenic indices, Anxiety

## Abstract

The multiple probiotic characteristics of strain TCI904 isolated in this study from natural fermented milk were investigated using a mouse model. TCI904 was identified as *Lactobacillus delbrueckii* subsp. *bulgaricu* (LDB*)*, a well-known lactic acid starter bacterium found in yogurt. TCI904 exhibited an outstanding pancreatic lipase inhibition activity among several strains of lactic acid bacteria in vitro*.* Its in vivo effects were further studied. In a comparison of mice fed a high-fat diet (HFD) and those fed a HFD combined with TCI904 for 9 weeks, differences were observed in various aspects of health, and the adverse effects of a HFD were prevented in the latter group. TCI904 effectively prevented fat and body weight accumulation without reducing food intake; it also modulated innate immunity and increased the level of IgA in feces, reversing the increased blood sugar and insulin levels and attenuated the hyperlipidemia caused by a HFD. Based on biochemical test data, compared with the HFD group, a HFD combined with TCI904 induced significant lowering of insulin resistance indicator, homeostasis model assessment-insulin resistance (HOMA-IR) and atherogenic indices of plasma (AIP), the atherogenic coefficient (AC) and cardiac risk ratio (CRR) and increased the cardioprotective index (CPI). In addition, the administration of TCI904 alleviated mood disorders caused by a HFD. Taking the recommended human dose of TCI904 did not affect the liver or kidney function, indicating that TCI904 has sufficient in vivo safety. Taken together, the results of the present study contributed towards validation of the probiotic benefits of lactic acid starter microflora. Orally taken TCI904 exhibited positive immune- and metabolic-modulating, and anxiolytic properties, especially in HFD-induced obesity.

**Supplementary Information:**

The online version contains supplementary material available at 10.1007/s13205-022-03356-3.

## Introduction

Obesity is the greatest health risk in the world, and many factors contribute to obesity. A prolonged and excessive intake of too many calories is the main factor, and millions of people are estimated to be at risk of cardiovascular disease and diabetes each year due to obesity (Wilding and Jacob 2021). Further, a high-fat diet (HFD) consumption increases depressive anxiety-like behaviors in both humans and animals (Sweeney et al. 2017). Although the underlying molecular mechanisms of the relationship between a HFD and psychiatric symptoms are unclear (Sweeney et al. 2017), presumably the most likely major factor is insulin resistance, which may affect insulin-sensitive pathways and subsequently neuronal activity (Liu et al. 2015). Depression status is also an independent risk factor for cardiovascular events and cardiovascular mortality (Smolderen et al. 2009).

In recent years, consumers have become fond of using dietary supplements to control weight gain without dieting, thereby promoting the development and production of health food. (Kearney 2010). Fermented milk is one of the major probiotic products commercialized globally, because the buffering capacity of fermented milk allows bacteria growth and survival (Guiné et al. 2020). Nowadays, most probiotics belong to the genera *Lactobacillus* and *Bifidobacterium* (Marissen et al. 2019); in fact, species belonging to the genera *Lactococcus* (Zhang et al. 2016), *Enterococcus* (Mansour et al. 2014) and *Propionibacterium* (Shu et al. 2013) are also considered probiotic bacteria. Moreover, *Streptococcus thermophilus* (Wu et al. 2021) and *Lactobacillus delbrueckii subsp. bulgaricus* (LDB) (Naidu et al. 1999) are considered to be potential probiotics, and *Lactobacillus* has long been used in fermented foods to promote human health (Chang et al. 2015; Faure et al. 2001).

A common market strategy today is to modulate the gut microbial composition and selectively stimulate the growth and activity of good bacteria by administering food supplements, and probiotics are a proven and effective method. Recent animal studies indicated that certain probiotics have potential as agents to treat or prevent obesity and overweight. *Lactobacillus* and *Bifidobacterium* are the most studied species in term of controlling body weight and exerting a positive impact on energy metabolism (Brusaferro et al. 2018). Importantly, and of particular note, different probiotic strains or different cultivations and varying preparation methods are likely to have different effects on obesity and obesity-related symptoms.

LDB has long been used as a lactic acid starter bacterium for fermentation products, but has not been considered to fully meet the definition of a probiotic in the past. Many recent studies have confirmed that this fermented bacterium possesses some probiotic functions and properties (Chou and Weimer 1999; Vinderola et al. 2002). The results of recent studies have suggested a beneficial impact of microbes of the genera *Akkermansia* and *Lactobacillus* on host lipid metabolism (Xu et al. 2020; Drissi et al. 2017). A previous study demonstrated potential probiotic, gene regulation and lipolysis effects of a cocktail of LDB and *Streptococcus thermophiles* (ST) in cultured differentiated 3T3-L1 cells (Guha et al. 2021). In another study, the addition of LDB to drinking water was shown to reduce the adverse effects of a high-cholesterol diet, but an effect on weight control was not obvious in the rat model (Hu et al. 2013). It was also shown that heat-killed *L. delbrueckii* LAB4 down-regulated the plasma cholesterol level through intraduodenal administration in 2-deoxy-d-glucose-induced hyperglycemic rats (Horii et al. 2019).

Although potential positive protective effects of various *L. delbrueckii* subspecies have been proposed, to the best of our knowledge, no comprehensive investigation has been performed to examine the versatile functions of LDB using an in vivo model. Despite a proposed immunity enhancement in elderly subjects by *L. delbrueckii* subsp. *bulgaricus* 8481 (Moro-García et al. 2013), we found scarce data in the literature related to the effects of LDB on weight control, blood sugar and lipid profile regulation or innate immunity modulation, in addition to the potential for cardiovascular disease prevention and insulin resistance improvement. Previous studies have shown differences in various aspects between the same species of different isolates (Jang et al. 2018; Yang et al. 2020; Kirjavainen et al. 1999).

In this study, we, therefore conducted an animal experiment to ascertain whether a single LDB alone exerted probiotic effects. TCI904, identified as LDB, was isolated from fermented milk in our laboratory. In an effort to identify a single probiotic strain that prevents obesity arising from an excessive calorie intake, the aim of the present study was to examine the beneficial effects of oral supplementation of probiotic TCI904 in mice fed a HFD. In addition to weight and fat control, we further examined the potential multiple health-promoting functions of TCI904, focusing on immune modulation, blood lipid profile adjustment, insulin-resistance improvement and other potential health benefits for the prevention of cardiovascular and psychiatric diseases. The results of this study may inform the formulation design of related health food products.

## Materials and methods

### Isolation and identification of TCI904 strain

An MRS plate containing 0.5% cysteine was directly coated with natural fermented milk and cultured in an anaerobic environment at 37 °C for 2 days, following which colonies formed on the plate were selected. A single colony was stored on a solid medium as a source for colony PCR, and lactic acid bacteria 16S rRNA and yeast internal transcribed spacer (ITS) were amplified by colony PCR. The PCR products were analyzed and compared using NCBI BLAST. The isolated bacterial strain was identified and confirmed to be *L. delbrueckii subsp. bulgaricus* (LDB), designated TCI904.

### Manufacturing process of TCI904 strain

Yeast peptone, glucose, MgSO_4_, NaCl, KH_2_PO_4_, K_2_HPO_4_, manganese gluconate and distilled H_2_O were mixed. After TCI904 was added, fermentation was carried out, following which a sample of the mixture was taken for analysis. After subsequent centrifugation, the bacterium mud was collected, water was added to the mix, and the bacteria were heated. The ensuing supernatant was then collected by centrifugation and filtered to sterilize. A freeze-drying protective agent was added to the mixture, which was then labeled and stored at 4 ℃.

### Pancreatic lipase activity inhibition

Pancreatic lipase is secreted by the pancreas and is a critical enzyme responsible for triacylglycerol and fat hydrolysis (Chen et al. 1994). The reduction of fat absorption through lipase inhibition is a reliable method to determine potential activity in terms of inhibition of dietary fat absorption and control of obesity (Ahn et al. 2012). *L. rhamnosus* GG (LF063), *L. delbrueckii* (LF006) and *Enterococcus faecium* (LF049) were isolated, identified and preserved in our laboratory. The frozen bacteria were activated and cultured on a plate at 37 °C under anaerobic conditions for 2 days. A single colony was selected for culture in 50 ml of MRS broth under anaerobic conditions for 2 days. The cultured conditioned supernatant was then collected by centrifugation for lipase activity determination. Lipase activity was assayed by modification of the method of Jang et al. (2008). Briefly, an enzyme buffer was prepared by the creation of a solution of porcine pancreatic lipase and Tris buffer (100 mM Tris-HC1 and 5 mM CaCl_2_, pH 7.0). Then, 25 μL of the sample was mixed with 25 μL of enzyme buffer and incubated for 15 min at 37 °C with 50 μL of the substrate solution (1.6 mM 4-nitrophenyl laurate). The enzymatic reactions were allowed to proceed for 30 min at 37 °C. Lipase activity was determined by measuring the hydrolysis of the substrate solution at 400 nm using an ELISA reader (BIO-TEK, Synergy HT, VT, USA). The percentage inhibition of enzyme activity was calculated as [(*A*_0_ – *A*_C_)/*A*_0_] × 100 (*A*_0_ = absorbance without sample, *A*_C_ = absorbance with sample).

### Animal experiment design

The complete procedure of the animal experiment is shown in Fig. 2A. The animal test protocol was approved by the Institutional Animal Care and Use Committee of the National Pingtung University of Science and Technology (NPUST-110-016). The mice were housed under a constant environment at 23 ± 1 °C and 55 ± 5% relative humidity with a 12 h dark–light cycle. They were allowed to eat and drink freely during the experiment, and all efforts were made to minimize animal suffering. A total of 32 male C57BL/6 mice aged 6 weeks were purchased from BioLASCO (Taipei, Taiwan). After 7 days of adaptation, the mice were randomly separated into the following four groups (*n* = 8 per group): normal diet (ND), HFD, HFD plus low-dose LDB (HFD + L) and HFD plus high-dose LDB (HFD + H). The HFD (Altromin C 1090-60; Lage, Germany) provided 60% kcal from fat and also contained 16% protein and 24% carbohydrates. TCI904 was administered daily at 10:00 am for 9 weeks by oral gavage. The HFD + L and HFD + H groups were fed 2 × 10^7^ CFU/kg and 10^8^ CFU/kg per day, respectively. Assessment of mouse behavior during the final week of feeding was performed. At the end of the experiment, the mice were killed with CO_2_ and dissected. Certain organs, epididymal fat, subcutaneous fat and perirenal fat were collected and weighed immediately, then the organs and blood were stored at − 80 °C or in formalin for further analysis.

### Immune organ index and immunoglobulin assay

The spleen and thymus weights of each necropsied mouse were measured. The immune organ index was calculated according to the following equation: immune organ weight (mg)/body weight (g) (Zhong et al. 2018). The level of fecal secretory immunoglobulin A (sIgA) was determined using a published method (Peters et al. 2004) with a mouse IgA ELISA detection kit (Biovision, Mountain View, CA, USA).

### Ex vivo spleen cell proliferation assay

The spleens were separated aseptically and minced in aseptic Hank’s balanced salt solution (HBSS) buffer, then passed through a sterilized cell strainer. The harvested splenocytes were freed of red blood cells by treatment with RBC lysis buffer (Thermo Fisher Scientific, Inc.) and resuspended in RPMI1640 complete medium. The collected cells were incubated in a 37 °C humidified incubator containing 5% CO_2_ for 6 h to remove adherent cells. Finally, the suspended cells were used as the splenocytes. Trypan blue exclusion was performed to check cell viability. 2 × 10^5^ cells per well were seeded onto a 96-well plate and incubated in the presence of mitogen lipopolysaccharide (LPS, 20 μg/ml) or concanavalin A (conA, 5 μg/ml) for 72 h, and cell proliferation was measured by MTT assay.

### Growth performance and feed efficiency ratio (FER)

Body weight and food intake were measured every 3 days using an electronic balance. The feed efficiency ratio (FER) was calculated as the ratio of body weight gain to the amount of food consumed (Chan et al. 2008). The percentage of fat mass to body weight was calculated by dividing the total weight of the subcutaneous fat, epididymal fat and perirenal fat by the final body weight.

### Sucrose preference test

Anhedonia-related behavior was determined using a two-bottle choice paradigm. Prior to testing, mice were habituated to the presence of two sucrose bottles on day 57, then one containing 1% sucrose and the other filled with water on day 58. After 12-h food and water deprivation, the mice were exposed to and accessed both water and 1% sucrose for 1 h on day 59. The sucrose preference percentage was defined as the consumed sucrose volume versus the total consumed volume (sucrose plus water) during the 1 h.

### Elevated plus maze (EPM) test

The activity of the mice in the EPM apparatus with two closed and two open arms was measured on day 62. The mice were placed in the center of the device, facing the open area and allowed to freely explore for five minutes. The maze was cleaned with 70% ethanol solution to remove any residual odors before every trial. A digital camera was positioned above and AnyMaze tracking system software was employed for analysis. Conventional measures were the cumulative time spent in and the number of entries into both open and closed arms. Data are presented as the percentage of time spent in open arms [(time in open arms/total time in any arm)] and of open-arm entries [(number of open-arm entries/total number of entries into any arm)]. To increase the sensitivity, the frequencies of ethological parameters including protected stretch-attend postures (pSAP), unprotected head-dipping (uHD) and rearing were measured. Behaviors were defined as unprotected when they were exhibited in the open-arm region of the EPM and protected if they occurred in a closed arm or in the central platform of the maze (Walf and Frye 2007). These indicators have been well validated to represent anxiety via the EPM (Fernandez Espejo 1997). Mice that were frozen for more than 40% of the time were excluded from the study (Chauke et al. 2012).

### Measurements of insulin, glucose and oral glucose tolerance test (OGTT)

Mice were fasted overnight prior to these tests on day 56 of the trial period. The fasting insulin level was assayed using a standard ELISA method (Voller et al., 1978) with a mouse insulin ELISA kit (Biovision). The homeostasis model assessment-insulin resistance (HOMA-IR) index was calculated using the following formula: (fasting glucose in mg/dL × fasting insulin in μU/mL)/405 (Choi et al. 2017). As positive associations have been confirmed between glucose intolerance and diabetes, hypertension and even the occurrence of cardiovascular disease (Kopelman 2000), thus, the OGTT was performed after overnight fasting. In the OGTT, the mice were administered 30% glucose solution at a dosage of 2 g/kg through intragastric gavage. Blood was taken every 30 min over a 2-h period and subjected to a glucose assay using a Roche Accu-Chek Mobile monitoring system.

### Histopathologic examination

Tissue samples were fixed by soaking in room-temperature neutral formalin solution (10%) for 24 h, dehydrated (70%, 85%, 95%, 100% alcohol, xylene) and embedded in paraffin. After embedding, the samples were cut into 3-μm sections and dewaxed, followed by staining with hematoxylin–eosin (H&E). Epididymal fat and liver tissues were immediately fixed with 4% formaldehyde for 24 h in situ, embedded in paraffin and cut into 4-μm sections for routine H&E staining. Specimens were viewed with a Nikon microscope and photographed at magnifications of 100 × and 400x.

### Biochemical analysis and atherogenic risk index

Serum was analyzed to measure total cholesterol, triglyceride, high-density lipoprotein cholesterol (HDL-C), aspartate aminotransferase (AST), alanine aminotransferase (ALT) and blood urea nitrogen (BUN) at the Veterinary Medical Teaching Hospital (National Pingtung University of Science and Technology, Pingtung, Taiwan). AST and ALT are indicators of liver function parameters, while BUN is an indicator of kidney function. Low-density lipoprotein cholesterol (LDL-C) and very-low-density lipoprotein cholesterol (VLDL-C) levels were calculated according to the formula reported in the previous literature (Friedewald et al. 1972). The atherogenic indices, including the atherogenic index of plasma (AIP), atherogenic coefficient (AC), cardiac risk ratio (CRR) and cardioprotective index (CPI), were calculated as according to the published literature (Orsolic et al. 2019) with formulas obtained from a previous study (Ilić et al. 2020) for calculation.

### Statistics

Data analysis was performed using SPSS software version 20 (IBM, Armonk, NY, USA) provided by our university. The data are expressed as the mean ± SEM. Tukey post-hoc analysis was performed following one-way ANOVA. *p* < 0.05 was considered to indicate a statistically significant difference.

## Results

### Inhibition of pancreatic lipase activity by TCI904

**Fig. 1:**
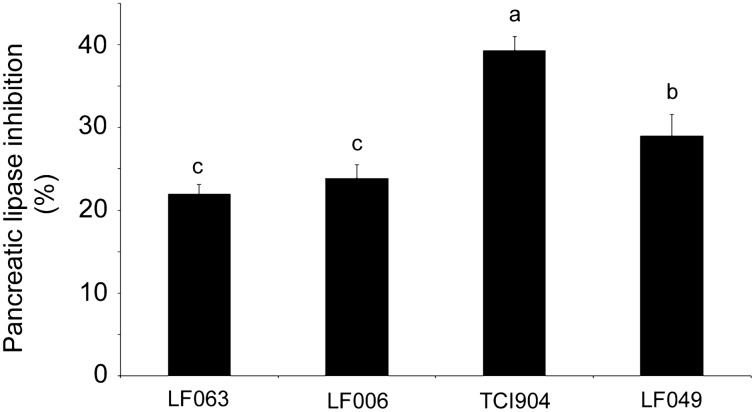
TCI904 possessed strong in vitro lipase inhibition activity. Various bacteria culture media were collected and analyzed as described in “Materials and Methods”. Values are the mean ± SEM of three independent experiments, each performed in duplicate. Bars marked with different letters indicate statistically significant differences among groups at *p* < 0.05

In the in vitro experiment, in comparison with known probiotics, TCI904 revealed the strongest pancreatic lipase inhibition activity (Fig. 1), suggesting that it has a potent suppressive effect on fat formation in vivo.

### TCI904 reduced body weight gain and fat pad deposition

**Fig. 2:**
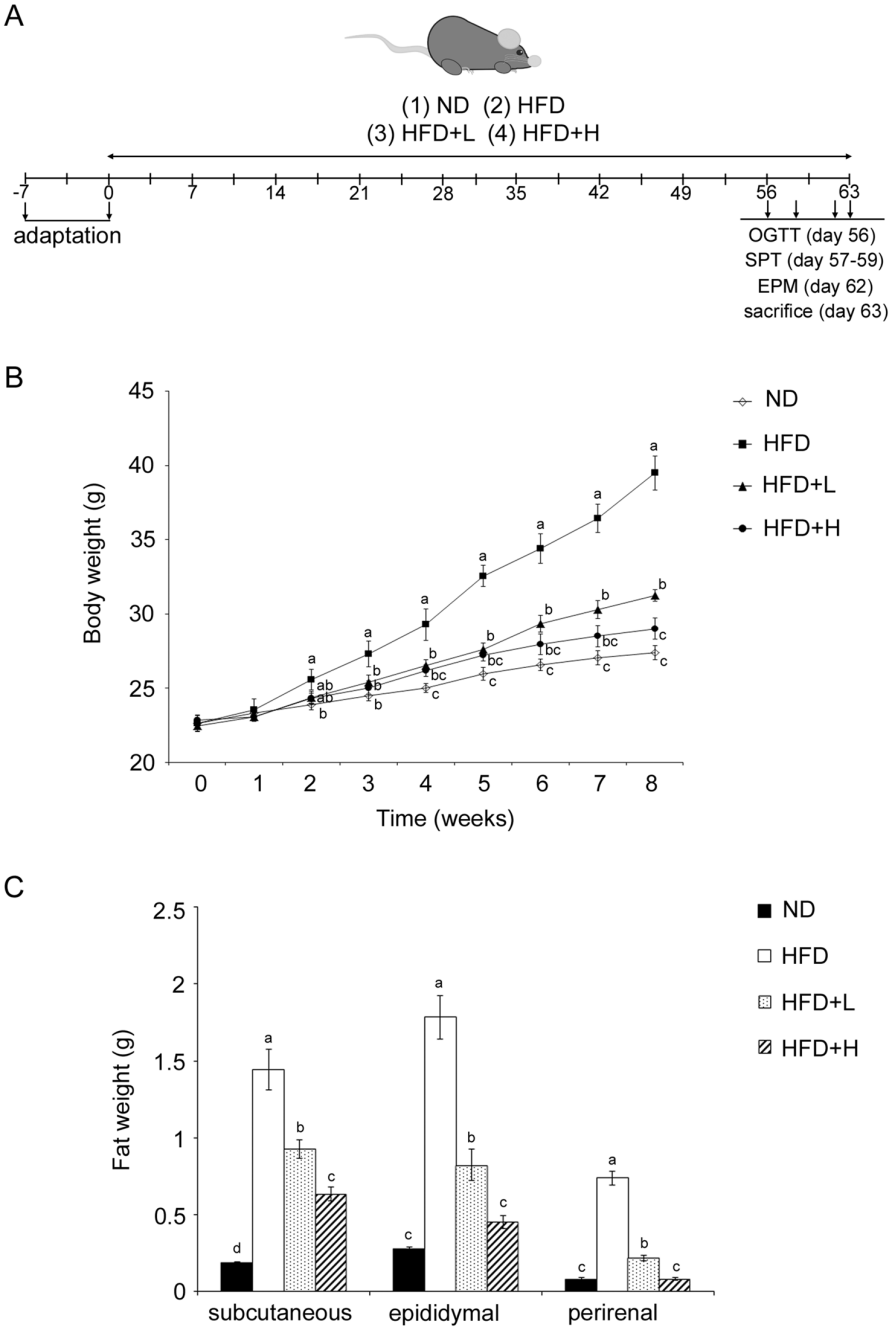

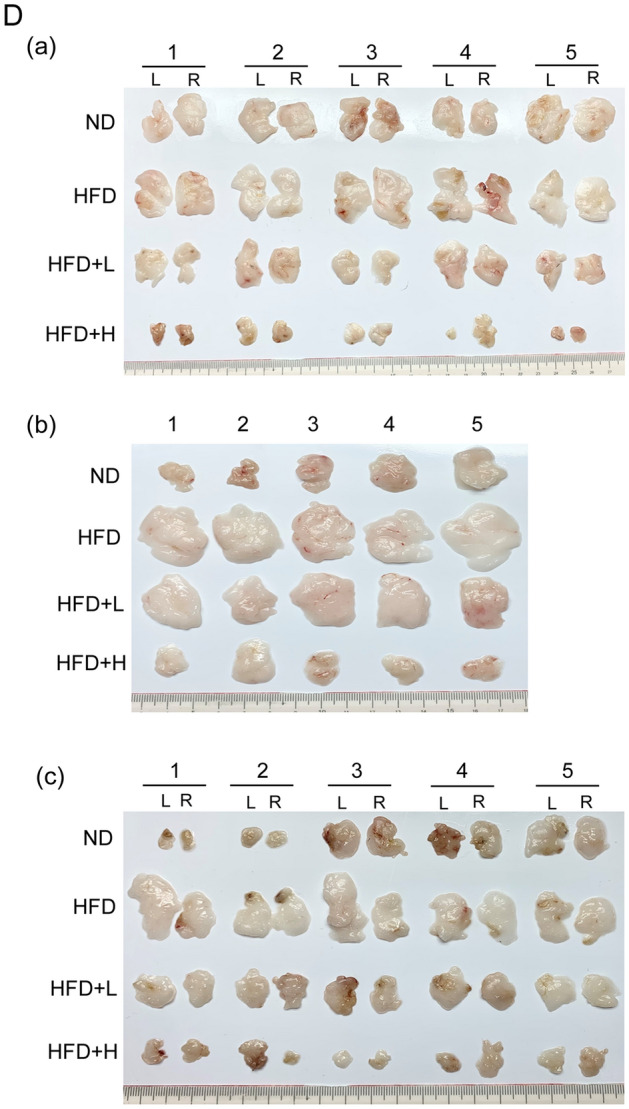
**A** Flowchart of animal experiments showing the time points at which assays were performed. **B** Effects of TCI904 on weekly body weight gain. Different letters at the same point represent significant differences at *p* < 0.05. **C** Fat tissues in mice administered a ND, HFD or TCI904-containing HFD were collected at the end of the trial for analysis of the average visceral fat mass. Results are presented as the means ± SEM. Bars marked with different letters indicate statistically significant differences among groups at *p* < 0.05. **D** In each group, five of the eight mice with a body weight in the middle range were selected, and certain visceral fat tissues were collected for comparison: (a) subcutaneous fat, *L* left side, *R* right side, (b) epididymal fat, (c) perirenal fat, *L* left side, *R* right side. *ND* normal diet, *HFD* high-fat diet, *HFD + L* HFD plus low-dose LDB, *HFD + H* HFD plus high-dose LDB

Over the whole course of the experimental period, none of the animals died, nor did they exhibit an abnormal food/water intake or visible behavioral changes. Comparing body weight between groups, the weight did not differ significantly between each group on the 7th day. Compared with the ND group, the HFD group showed a significant weight gain after 2 weeks of a HFD and dramatic, rapid weight gain from the 3rd week until the end of the trial period. After 8 weeks of the trial period, the weight gain in the ND animals was 21.11%, and that in the HFD group was 74.59%, whereas the body weight gain in the mice fed low- and high-doses of TCI904 was 39.22% and 27.05%, respectively. After the 8-week trial period, the weights of the HFD and TCI904 co-fed mice were slightly higher than that of the ND mice; the body weight of the high-dose TCI904-fed mice returned to the level of the ND mice. Thus, consuming TCI904 for only 2 weeks showed a partial weight-control effect. A longer ingestion duration of TCI904 had a more significant effect on controlling weight gain in a dose-dependent manner, a higher dose of TCI904 exerting a better effect and long-term rendering a return to a normal weight (Fig. 2B). There were no differences in the amounts of food and water consumed in each group of mice during the experimental observation period (data not shown). Thus, appetite was unchanged in mice with or without TCI904 administration. The FER indicates the ability of an animal to convert each unit of food into body weight. Although the food intake of each group of mice was the same, the FER of the HFD mice was higher than that of the ND mice. It is reasonable to speculate that the energy metabolism of the mice on a ND and a HFD must be significantly different. TCI904 feeding could affect the FER, and a HFD combined with a higher dose of TCI904 had no effect on the FER in comparison with the ND mice (Table 1, column 1).

**Table 1:** Feed efficiency ratio (FER), growth, innate immunity and other indicators of the mice in each group

	FER	Fat mass (% of body weight)	Thymus index	Spleen index	IgA fecal (μg/mg feces)	brain/body weight (ratio)
ND	0.02 ± 0.01^c^	5.47 ± 3.93^c^	1.81 ± 0.14^a^	4.36 ± 0.42^bc^	100.38 ± 8.25^a^	0.95 ± 0.03^a^
HFD	0.03 ± 0.02^a^	19.56 ± 2.42^a^	0.65 ± 0.08^d^	5.79 ± 0.29^a^	49.88 ± 7.59^c^	0.62 ± 0.01^b^
HFD + L	0.04 ± 0.02^b^	12.42 ± 1.97^b^	1.19 ± 0.14^c^	4.97 ± 0.49^b^	73.75 ± 4.94^b^	0.83 ± 0.03^a^
HFD + H	0.04 ± 0.03^c^	7.46 ± 1.21^c^	1.51 ± 0.18^b^	4.26 ± 0.47^c^	93.75 ± 7.82^a^	0.92 ± 0.04^a^

Feed efficiency ratio (FER) = body weight gain (g/day)/food intake (g/day)

Data represented as mean ± SEM, *n* = 8 per group. Different superscript in the same column represent significant differences at p < 0.05

*ND* normal diet, *HFD* high-fat diet, *HFD + L* HFD plus low-dose LDB, *HFD + H* HFD plus high-dose LDB

At the end of the experiment, subcutaneous fat, epididymal fat and perirenal fat were collected, weighed and photographed individually. The data indicated that a HFD caused greater accumulation of fat in the body and a higher fat mass as a percentage of body weight; consuming TCI904 had a significant effect in terms of inhibition of body fat formation, and a higher dose induced a noticeable effect (Table 1, column 2 and Fig. 2C). The collected fat from each part of the mouse in each group is shown in Fig. 2D; this clearly shows the differences in fat mass and volume.

### TCI904 modulated the HFD-induced skewed immune response

**Fig. 3:**
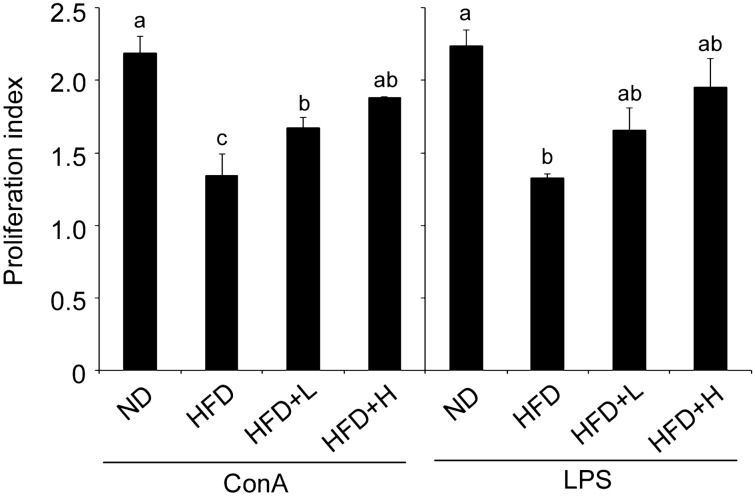
Effects of HFD and TCI904 on proliferation of spleen lymphocytes isolated from mice spleen. Cells were co-cultured with 5 μg/ml concanavalin A (ConA) or 20 μg/ml lipopolysaccharide (LPS) for 72 h, and cell proliferation was measured by MTT assay. The OD value at 570 nm was measured and results are expressed as the ratio of non-stimulated cells. Bars marked with different letters indicate statistically significant differences among groups at *p* < 0.05. *ND* normal diet, *HFD* high-fat diet, *HFD + L* HFD plus low-dose LDB, *HFD + H* HFD plus high-dose LDB

sIgA is the predominant antibody isotype secreted into the small intestine, which serves as the first line of defense for intestinal mucosal immunity. A previous study showed that a HFD decreased the sIgA coating gut microbiota, changed the microbial composition and affected certain metabolites (Muhomah et al. 2019). Furthermore, sIgA production in feces is dependent on the bacterial composition in the gut. In addition to measuring the level of sIgA in feces, we also measured the weights of lymphoid organs thymus and spleen, then determined the immune organ index. The thymus and spleen organ index can reflect the strength of the body’s immune function to a certain extent. At the end of the experiment, the level of fecal sIgA tended to be decreased in the HFD mice, while the suppression of sIgA induced by a HFD was almost completely reversed by feeding with a high dose of TCI904 (Table 1, column 5). The HFD-induced thymus atrophy and spleen hypertrophy were ameliorated by oral TCI904 administration, and a higher dose was more effective (Table 1, columns 3 and 4). To confirm the proliferative activity of the lymphocytes, we subsequently performed an ex vivo splenocyte proliferation assay for each group. Both T cell and B cell proliferation were significantly lower in the HFD group than the ND group. The lymphocyte stimulation effects of TCI904 treatment combined with ConA and LPS are shown in Fig. 3. Therefore, TCI904 is a promising immunomodulatory probiotic strain.

### TCI904 alleviated insulin resistance caused by a HFD

**Fig. 4:**
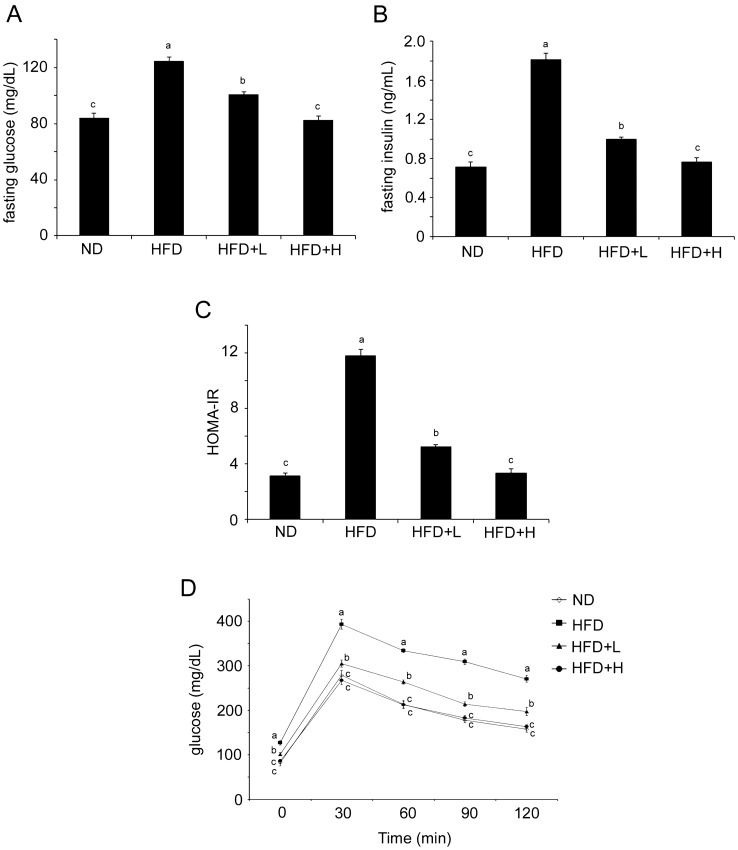
Effects of TCI904 supplementation on glucose and insulin sensitivity in HFD-fed mice. **A** Fasting glucose, **b** fasting insulin, **C** HOMA-IR, **D** OGTT in week eight. The HOMA-IR index was calculated as described in “Materials and Methods”, and the OGTT was performed after overnight fasting. Data are presented as means ± SEM. Bars marked with different letters indicate statistically significant differences among groups at *p* < 0.05. *ND* normal diet, *HFD* high-fat diet, *HFD + L* HFD plus low-dose LDB, *HFD + H* HFD plus high-dose LDB

Insulin is a hormone produced by the pancreas to control the blood glucose level and lipid homeostasis (Saltiel and Kahn 2001). Thus, the serum insulin and glucose levels were measured. The current experimental data revealed higher fasting glucose and fasting insulin levels in the HFD mice as compared with the ND group (Fig. 4A and B), and such changes were reflected in the increase in the HOMA-IR index (Fig. 4C), while the TCI904-treated group showed positive regulatory effects. More advanced positive effects were observed in the return to baseline, with no difference from the ND group being seen in the TCI904 higher dose-fed group (Fig. 4). Figure 4D shows a glucose peak at 30 min in all mouse groups, which then gradually decreased with time. A HFD kept blood glucose at a high level and caused it to reduce much more slowly than in the other groups. TCI904 improved the impaired sensitivity to glucose induced by a HFD, and the change curve of glucose concentration in the high-dose TCI904-fed mice was restored to the same as that in the ND mice.

### TCI904 improved lipid profile and attenuated HFD-induced hyperlipidemia

Hyperlipidemia medications currently used in clinical applications have side effects including liver and kidney toxicity, muscle symptoms and rhabdomyolysis (Thompson et al. 2003). Therefore, several new alternatives have attracted attention in recent years. To evaluate the potential hypolipidemic effect of TCI904, the serum lipid profile was analyzed at the end of the experimental period. The results are shown in Table 2 and indicated that a HFD significantly increased the serum total cholesterol, triglyceride and LDL-C levels as compared with the ND mice, the adverse effects of a HFD being very obvious. TCI904 feeding significantly reduced these markers of hyperlipidemia in a dose-dependent manner as compared with the untreated HFD mice. In contrast, the HDL-C level was significantly elevated following TCI904 treatment as compared with the HFD-fed mice. The serum AST, ALT and BUN levels were not altered in any group (Table 2), thus confirming that the liver and kidney function was normal until the end of the experiment, and the dose of TCI904 was of a safe concentration. Furthermore, AIP, AC, CRR and CPI were calculated from the biochemical lipid parameters. AC, AIP and CRR were significantly elevated, while CPI was decreased in the HFD mice as compared with the ND mice. The highest AIP, AC and CRR, and the lowest CPI value were seen in the HFD group (Table 3). Both low and high doses of TCI904 tended to render AIP, AC and CRR nearer to those in the ND mice in a dose-dependent manner. CPI was significantly reduced in the HFD mice as compared with the ND mice, and a high dose of TCI904 increased the CPI value. Taken together, these findings strongly suggested that TCI904 may ameliorate HFD-mediated hyperlipidemia and modify the blood lipid balance towards a healthy direction.

**Table 2:** Influence of TCI904 on the concentration of various clinical serum parameters in each mice groups

	TG (mg/dL)	TC (mg/dL)	HDL-C (mg/dL)	LDL-C (mg/dL)	AST (U/L)	ALT (U/L)	BUN (mg/dL)
ND	77.13 ± 11.35^d^	82.88 ± 7.7^c^	42.38 ± 4.24^a^	25.08 ± 11.04^c^	47.4 ± 6.86^a^	29.8 ± 4.58^a^	24.46 ± 2.19^a^
HFD	263.13 ± 18.37^a^	217 ± 13.15^a^	23.13 ± 2.37^c^	141.25 ± 12.36^a^	48.5 ± 5.44^a^	28.5 ± 7.27^a^	20.23 ± 1.81^a^
HFD + L	165.25 ± 12.93^b^	147.13 ± 11.2^b^	31.5 ± 1.8^b^	82.58 ± 10.23^b^	43.6 ± 2.15^a^	23.8 ± 3.54^a^	19.74 ± 2.13^a^
HFD + H	100.25 ± 8.07^c^	92.25 ± 5.47^c^	39.5 ± 3.35^a^	32.7 ± 7.87^c^	48.6 ± 3.07^a^	28.2 ± 2.99^a^	24.24 ± 7.28^a^

Data represented as mean ± SEM, *n* = 8 per group. Different superscript in the same column represent significant differences at *p* < 0.05

*ND* normal diet, *HFD* high-fat diet, *HFD + L* HFD plus low-dose LDB, *HFD + H* HFD plus high-dose LDB, *TG* triglyceride, *TC* total cholesterol, *HDL-C* high-density lipoprotein cholesterol, *LDL-C* low-density lipoprotein cholesterol, *AST* aspartate aminotransferase, *ALT* alanine aminotransferase, *BUN* blood urea nitrogen

**Table 3:** Effects of TCI904 on insulin resistance and cardiovascular risk indices

	AIP	AC	CRR	CPI
ND	0.26 ± 0.06^d^	0.99 ± 0.34^c^	1.99 ± 0.34^c^	1.84 ± 0.74^a^
HFD	1.06 ± 0.05^a^	8.44 ± 0.65^a^	9.44 ± 0.65^a^	0.16 ± 0.02^b^
HFD + L	0.72 ± 0.06^b^	3.68 ± 0.28^b^	4.68 ± 0.28^b^	0.39 ± 0.44^b^
HFD + H	0.41 ± 0.06^c^	1.36 ± 0.28^c^	2.36 ± 0.28^c^	1.31 ± 0.44^a^

Data represented as mean ± SEM, *n* = 8 per group. Different superscript in the same column represent significant differences at *p* < 0.05

*ND* normal diet, *HFD* high-fat diet, *HFD + L* HFD plus low-dose LDB, *HFD + H* HFD plus high-dose LDB

*AIP* atherogenic indices of plasma, *AC* atherogenic coefficient, *CRR* cardiac risk ratio, *CPI* cardioprotective index

### TCI904 reduced hepatic steatosis and adipocyte size in mice fed a HFD

**Fig. 5:**
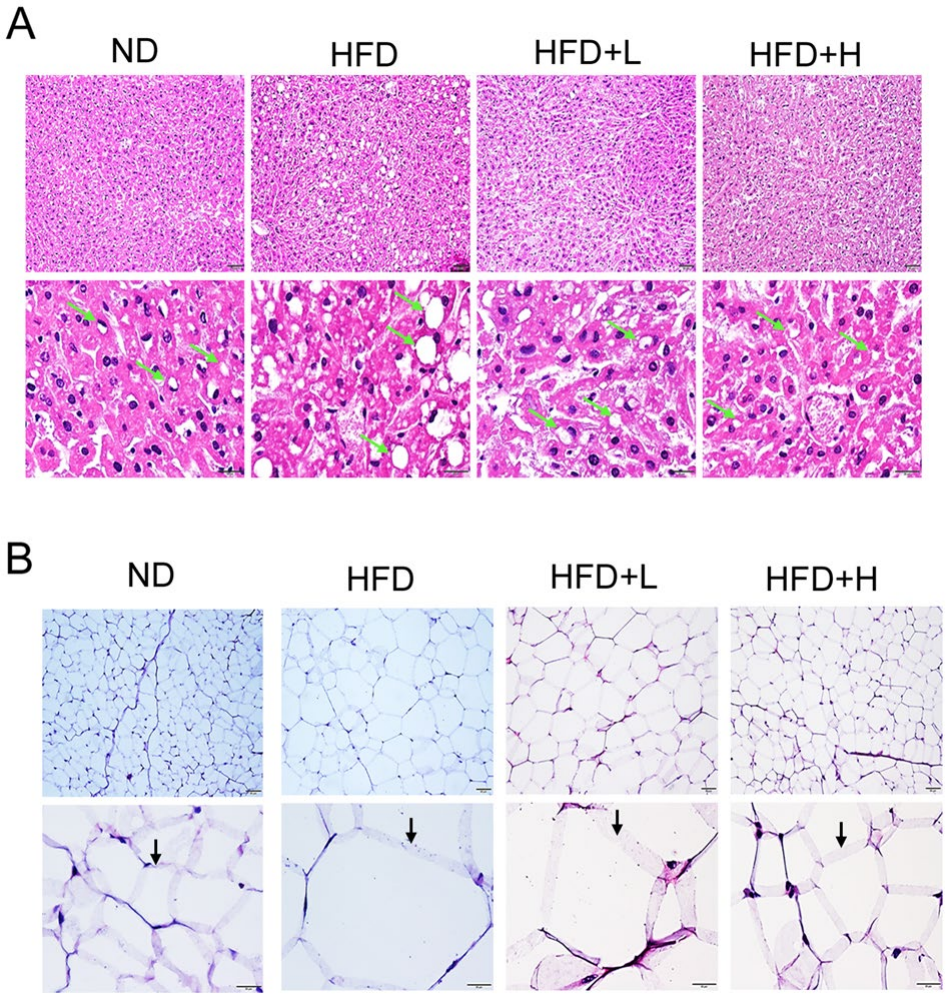
Effects of HFD and TCI904 on liver and adipocyte morphologic characterization. HE staining of histological sections of **A** liver (white areas pointed to by arrows indicate fat), **B** epididymal adipocytes (lower panel of magnified images shows enlarged fat cells with a HFD). Upper panel, magnification at 100×, scale bars: 50 μm. Lower panel, magnification at 400×, scale bars: 20 µm. *ND* normal diet, *HFD* high-fat diet, *HFD + L* HFD plus low-dose LDB, *HFD + H* HFD plus high-dose LDB

A HFD most directly and significantly affects the liver and adipose tissue. As shown in Fig. 5A, the HFD caused significant numerous fat vehicle accumulation in the liver as compared with the ND group, which characterizes typical hepatic steatosis. The HFD-fed group demonstrated an increased adipocyte size as compared with the ND group (Fig. 5B). Nevertheless, treatment with TCI904 markedly reduced the fat accumulation in liver parenchyma and the size of adipocytes, and the effect of a high dose of TCI904 was more pronounced.

### Anxiolytic effects of TCI904 supplementation in HFD-induced obese mice

**Fig. 6:**
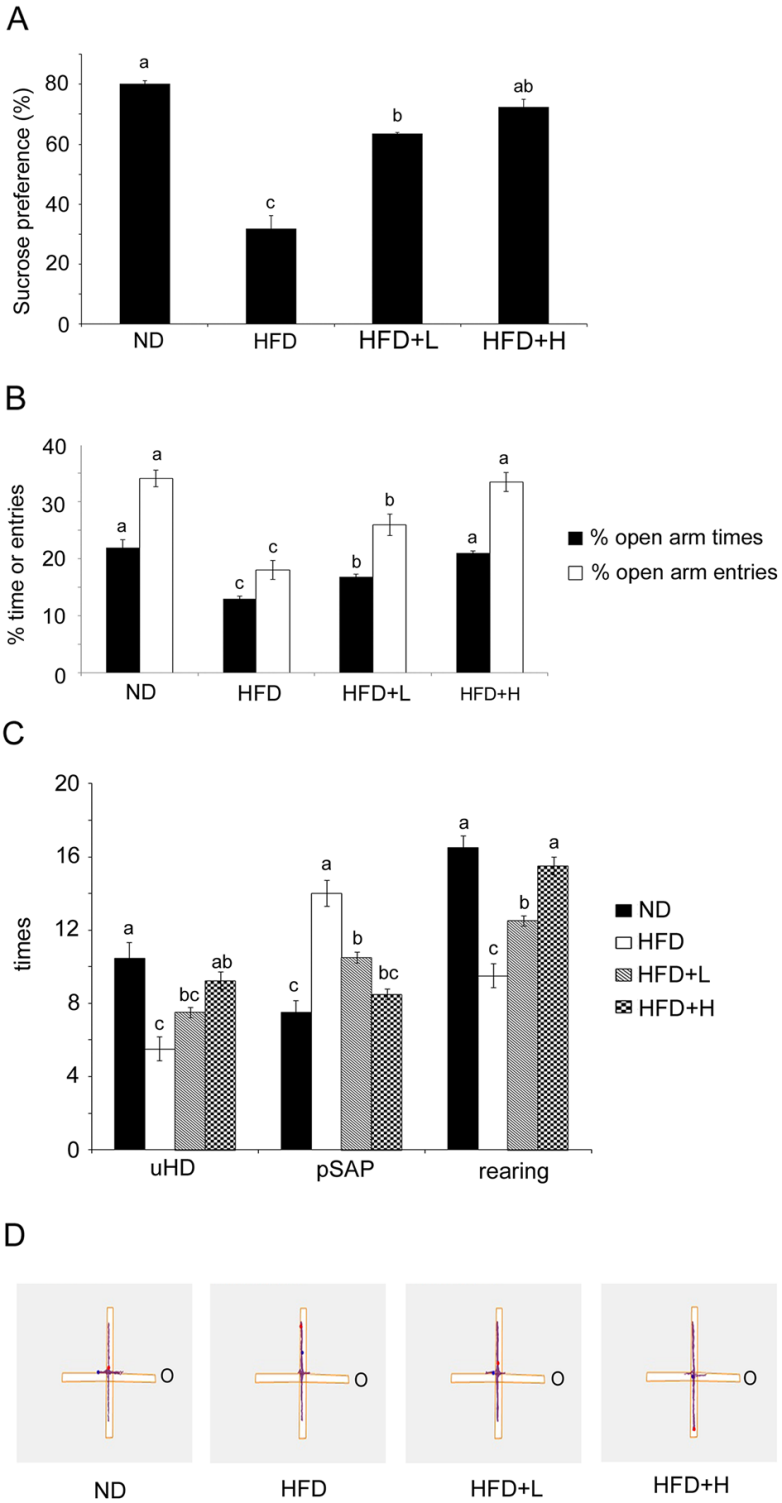
Comparisons of depression and anxiety in the experimental groups. **A** Sucrose preference test and **B** % time spent in and number of entries into open arms of the EPM as indices of anxiety level. **C** Frequency of uHD, pSAP and rearing posture of mice in the EPM test, showing the effects of TCI904 on animal behavior. Data are presented as the means ± SEM. Bars marked with different letters indicate statistically significant differences among groups at *p* < 0.05. **D** Trajectories of the mice in each group in the EPM within 5 min, in which darker red trajectories indicate more frequent locations of the animals. (O: open arm.) *ND* normal diet, *HFD* high-fat diet, *HFD + L* HFD plus low-dose LDB, *HFD + H* HFD plus high-dose LDB

Although brain weights were unchanged (data not shown), the ratio of brain to body weight was dramatically decreased in the HFD-fed mice as compared with the ND mice. The ratio difference was reversed by TCI904 feeding (Table 1, column 6). After feeding for almost 9 weeks, as compared with the ND mice, the HFD-treated mice exhibited a significantly reduced motivation to pursue the pleasure of sucrose, which is considered an anhedonia-like symptom (Fig. 6A). Figure 6B illustrates that HFD consumption increased anxiety, which can be seen from the percentage decrease in time spent in and number of entries into the open area of the EPM. At either a low or high dose, the TCI904-treated mice exhibited an increased sugar over water preference and entered the open arm more frequently and stayed longer in the EPM. Observing mouse postures in different areas of the EPM can enable more accurate determination of mouse emotions. It was observed that as compared with the control ND mice, the HFD mice exhibited low frequencies of uHD and rearing, but more frequent pSAP. The mice that received TCI904 had significantly higher frequencies of uHD and rearing and fewer pSAP as compared with the HFD mice (Fig. 6C). Figure 6D shows the mouse walking tracks in the EPM and indicates that mice with higher anxiety levels had significantly fewer trajectories into the open areas. Although the mechanism is not fully understood, our current results were consistent with previous research, showing that a HFD increases anxious behavior in mice as compared with the ND control group, and TCI904 supplementation reduced depressive anxiety associated with a HFD.

## Discussion

The causes of obesity are complex, but the main factor is an excessive calorie intake generated from a high-sugar, high-fat or high-cholesterol diet. Critically, maternal HFD-induced obesity has significant detrimental and adverse effects in offspring (Johnson et al. 2017). Obesity may also be linked not to diet but to immunity, metabolic imbalances and psychiatric disorders. No matter what factors lead to obesity, it is easy to induce hyperglycemia, hypertension or hyperlipidemia, which greatly increase the risks of multiple diseases (Hariharan et al. 2022). Weight loss through exercise or a diet plan is ineffective and not sustainable, and prescription weight loss pills not only have restrictions on their use, but also have many side effects. Thus, there is an urgent need to identify a healthy way to control weight by suppressing adipose tissue formation under a general lifestyle without dieting. Probiotics are considered to be of the greatest potential (Benioudakis et al. 2021). LDB isolated from fermented yogurt in our laboratory showed the potential to be applied in this regard, with the hope of achieving health benefits through the use of a single strain, which was the specific aim of the present study.

TCI904 inhibited body weight gain by reduction of fat formation in a dose-dependent manner in HFD-treated mice. This effect was not associated with food intake, as no food consumption difference was observed between any mouse groups, suggesting that TCI904 may not affect appetite. Rather, it affects food conservation and the metabolic rate, although the underlying mechanism is not clear. We speculated that TCI904 affects multiple mechanisms through indirect means.

It is already well known that insulin sits at the critical center of metabolic disorders, including obesity, diabetes, hyperlipidemia, non-alcoholic fatty liver disease and all risk factors of cardiovascular disease (Kato et al. 2015; Oseini and Sanyal 2017). A HFD causes insulin resistance and glucose intolerance, which are the most critical risk factors for diabetes. Numerous studies have demonstrated the effects of different probiotics in this regard. Previous study showed probiotic mixtures containing various *Bacillus* sp. or *Bacillus* combined with fermented soybean pastes to prevent HFD-induced obesity and insulin resistance in mice (Kim et al. 2018a, b). Certain single *Bacillus* or *Lactobacillus* strain administration, for example, *Bacillus licheniformis* (Cao et al. 2019), *Bacillus amyloliquefaciens* (Wang et al. 2019), *Lactobacillus casei* (Naito et al. 2011) and *Lactobacillus plantarum* (Kim et al. 2018c), have also shown efficacy. Unlike these prior studies, the present study utilized a single LDB strain; there have been no previous studies on the efficacy of this bacterium in relation to weight control-related symptoms using a mouse model.

A human trial confirmed that the presence of yogurt bacteria *Streptococcus thermophilus* and LDB in the feces of individuals who ate yogurt indicated that the two strains of bacteria can survive and pass through the gut (Mater et al. 2005). Another study administered inactivated LDB through the intraduodenal route to rats, following which the sympathetic nerve activity in the rats was reduced, and the blood sugar level was lowered. Therefore, regulation of nerve conduction may be one of the modes of action of probiotics (Horii et al. 2019). Previous studies have mentioned that probiotics have no significant effect on weight gain caused by a high-cholesterol diet, but can regulate the transcription of genes related to the liver and lipid metabolism (Hu et al. 2013). Animal studies have emphasized that obesity is associated with adipose tissue inflammation and oxidative stress in the liver, and certain *Lactobacillus* strains can reduce the transcription of proinflammatory cytokine genes, such as IL-6, TNF-α and IL-1β (Park et al. 2013). Study employing a cultured 3T3-L1 system indicated that LDB combined with an ST cocktail regulates the peroxisome proliferator-activated receptor gamma (PPAR-γ) gene involved in adipocyte maturation and genes related to the fatty acid translocator, such as adipocyte fatty acid binding protein (AP2) (Wang et al. 2021). Thus, numerous genes, enzymes and mechanisms are linked to reversing symptoms related to a HFD. In fact, it is currently difficult to fully explore this issue. The FER indicates the ability to convert food into body weight, and there were no differences in the amount of food consumed by the various groups of mice in our experiment. This indicated that HFD-induced obesity is caused by receiving too many calories in a regular lifestyle. Whether or not related metabolic genes are regulated by TCI904 requires further exploration.

The immune effect of LDB has not been reported previously. Our study showed for the first time that LDB can boost innate immunity. The thymus is the primary immune organ that allows T lymphocyte maturation. The spleen is the largest lymphoid organ and the most important secondary lymphoid organ that is responsible for filtering out pathogens and maintaining lymphocytes. Both organs change in size due to immune status, and their weight can be used as an immune indicator. The thymus shrinks with age, and a HFD can accelerate this phenomenon; additionally, splenomegaly has been observed in HFD-fed mice (Lee et al. 2017). A previous study reported that a HFD impairs the ability of dendritic cells to facilitate T cell expansion and may in turn reduce differentiation of IgA-producing cells (James et al. 2012). Numerous pieces of evidence over the past few years point to the ability of sIgA to influence the composition of gut microbiota, to uptake and deliver antigens to immune cells in the gut and to modulate the gut's inflammatory response to pathogens or allergens (Mantis et al. 2011). Yang et al*.* previously discovered that different *Bacteroides ovatus* strains induce various fecal IgA levels (Yang et al. 2020). Taking the results of these studies together, probiotic strains are key players. In the present study, TCI904 isolated in our lab caused macroscopic changes in the thymus and spleen, which were reflected in the organ weights, and simultaneously increased the concentration of IgA antibodies in the stools, indicating that TCI904 can activate innate immunity. Intestinal IgA and macrophages are critical components in the gut barrier and mediators in the intestinal immunity balance (Sheikh and Plevy 2010). Fecal IgA production depends on colonization by gut microbiota. Whether or not TCI904 can affect the systemic immune system by linking with local immunity, or plays a possible positive role in maintaining gut microbial composition, requires further investigation.

Epidemiological studies have indicated that obesity, fat accumulation, dyslipidemia and glucose intolerance are highly positively associated with cardiovascular disease risk. Insulin resistance is the cause and cure of metabolic syndrome and its complications, including hypertension, dyslipidemia, type 2 diabetes and gout, and also induces cardiovascular disease, early heart disease, fatty liver, and even cancer (Andreadi et al. 2022). High-fat diets are known to have a number of negative effects, including easily visible obesity and weight gain, as well as blood sugar increases and dyslipidemia that require testing to detect. Insulin resistance is common in obesity, which means that the sensitivity of the liver, muscles, and fat cells to insulin is reduced. The glucose in the blood cannot enter the cells for decomposition and energy supply. Although the true cause of insulin resistance remains undetermined, it has been clinically demonstrated that energy accumulation is an important factor. The assessment of insulin resistance can effectively detect the occurrence of prediabetes and monitor the development of diabetes. Methods of assessing insulin resistance exist, such as the Pancreatic Suppression Test, Insulin Suppression Test, Glucose Clamp, and Hyperinsulinemic-Euglycemic Clamp Technique (Gutch et al. 2015; Pratt-Phillips et al. 2015). However, these methods are not only complex, time-consuming, and expensive, but also not practical in clinical practice and epidemiology. Therefore, a variety of simple and indirect assessment formulas have been developed clinically. In 1985, the HOMA-IR index was proposed by Matthews et al., using which the degree of insulin resistance can be obtained by calculating the easily obtained fasting insulin and fasting blood glucose values (Matthews et al. 1985). Insulin resistance can be used to identify patients with a normal body mass index (BMI) and a slim body but high visceral fat and a potential diabetes risk. Our data showed that the blood sugar and insulin levels were reduced significantly in the TCI904-treated mice. The action mechanism for TCI904-mediated sugar-lowering has not been explored, but reasonable speculation is that it is likely to be related to pancreatic lipase inhibition by TCI904 (Fig. 1).

The fats in the blood are mainly cholesterol and triglycerides. Cholesterol can be roughly divided into two categories, LDL-C and HDL-C. It is generally believed that LDL-C will adhere to the blood vessel wall, while on the other hand, HDL-C will transport cholesterol from peripheral tissues to the liver for metabolism, which has a protective effect on blood vessels. However, a lower cholesterol level is not always better; it also depends on the ratio of triglycerides and high- to low-density cholesterol. In some cases, the cholesterol level may be normal, but blood lipids may still be abnormal. Therefore, through the characteristics of different blood fats, experts have further designed some different indicators, those commonly used being the AIP, AC, CRR and CPI, to understand the risk of atherosclerosis. The greater the increase in the AIP, AC and CRR, the higher the risk of CVD (Orsolic et al. 2019). The current study data were consistent with the calculated atherogenic indicators: the AIP, AC and CRR were attenuated in the TCI904 intervention group, whereas the CPI was highly improved following TCI904 treatment.

It is well known that a HFD plays a causative role in the development of depression and anxiety (Tan and Norhaizan 2019), so we next assessed whether TCI904 improved the depressive anxiety behavior of the mice by sucrose consumption testing and the elevated plus maze (EPM). The current study confirmed for the first time that LDB TCI904 probiotics strain reduced anxiety behaviors caused by a HFD. In this context, a HFD is known to cause circulating fatty acid overproduction and trigger oxidative stress and a systemic inflammatory response in the body (Tan and Norhaizan 2019). Immune cells and circulatory cytokines, as well as free fatty acids, reach the brain, such as the hypothalamus, prefrontal cortex, and amygdala, which may affect multiple brain areas and change neurochemical signaling, directly or indirectly mediating anxiety and anhedonia (Dutheil et al. 2016).

From the above results, we concluded that TCI904 exhibited great potential as a safe and potent antihyperglycemic, antihyperlipidemic and anxiolytic agent against the effects of a HFD. Oral administration of TCI904 acts through the regulation of food utilization and individual metabolism and may play a role in ameliorating metabolic, cardiovascular and psychiatric disorders. Although the underlying health-promoting mechanisms of TCI904 are not well understood, at least in part, the critical action mechanism of TCI904 is immunomodulation, thus bringing about multiple beneficial health effects. Further research on the molecular action mechanisms underlying the effects of TCI904 would facilitate health food product commercialization.

## Conclusion

In this study, we isolated TCI904, a *L. delbrueckii* subsp. *bulgaricus* (LDB) strain, which was previously considered as a lactic acid starter bacterium, and confirmed that it has the characteristics of a probiotic. The results of our in vivo mouse model indicated that the most significant effect of TCI904 was inhibition of fat formation in the mice due to a HFD, and prevention of fat and body weight accumulation by TCI904 acts through regulating metabolism without requiring a reduced food intake. The fecal IgA level and other biochemical tests revealed that TCI904 could reduce the risk of cardiovascular disease induced by a HFD. However, this study could not provide a more precise description of how TCI904 affects metabolism in relation to a HFD, such as the mechanisms involved. First, we do not know whether introducing TCI904 may modify the gut microbiota composition, which may in turn play roles in anti-obesity and other beneficial effects, and the directly associated genes remain to be identified. Second, it is unclear whether TCI904 affects neurotransmission in the amygdala, which is responsible for the emotional response. Taken together, we demonstrated that TCI904 may improve immunity and metabolic syndrome in a mouse model of obesity due to a HFD, and can assist in weight control and exhibit an anxiolytic effect, especially in HFD-induced obesity.

## Supplementary Information

Below is the link to the electronic supplementary material.

Supplementary file1 (DOCX 61 KB)

## Data Availability

All data have been included in the manuscript.
