# Supplementary material for: Lactobacillus delbrueckii subsp. bulgaricus strain TCI904 reduces body weight gain, modulates immune response, improves metabolism and anxiety in high fat diet-induced obese mice
Source: 3 Biotech. 2022 Nov 4;12(12):341. doi: 10.1007/s13205-022-03356-3 (PMC9636364; doi:10.1007/s13205-022-03356-3)
Supplement: Supplementary file 1 — Supplementary file1 (DOCX 61 KB) [file 13205_2022_3356_MOESM1_ESM.docx]

**Molecular Identification Report of TCI904**

16S rRNA Sequence: AGGCCTTGGCGGGCGGGGCTAATACATGCAGTCGAGCGAGCTGAATTCAAAGATCCCTTCGGGGTGATTTGTTGGACGCTAGCGGCGGATGGGTGAGTAACACGTGGGCAATCTGCCCTAAAGACTGGGATACCACTTGGAAACAGGTGCTAATACCGGATAACAACATGAATCGCATGATTCAAGTTTGAAAGGCGGCGTAAGCTGTCACTTTAGGATGAGCCCGCGGCGCATTAGCTAGTTGGTGGGGTAAAGGCCTACCAAGGCAATGATGCGTAGCCGAGTTGAGAGACTGATCGGCCACATTGGGACTGAGACACGGCCCAAACTCCTACGGGAGGCAGCAGTAGGGAATCTTCCACAATGGACGCAAGTCTGATGGAGCAACGCCGCGTGAGTGAAGAAGGTTTTCGGATCGTAAAGCTCTGTTGTTGGTGAAGAAGGATAGAGGCAGTAACTGGTCTTTATTTGACGGTAATCAACCAGAAAGTCACGGCTAACTACGTGCCAGCAGCCGCGGTAATACGTAGGTGGCAAGCGTTGTCCGGATTTATTGGGCGTAAAGCGAGCGCAGGCGGAATGATAAGTCTGATGTGAAAGCCCACGGCTCAACCGTGGAACTGCATCGGAAACTGTCATTCTTGAGTGCAGAAGAGGAGAGTGGAATTCCATGTGTAGCGGTGGAATGCGTAGATATATGGAAGAACACCAGTGGCGAAGGCGGCTCTCTGGTCTGCAACTGACGCTGAGGCTCGAAAGCATGGGTAGCGAACAGGATTAGATACCCTGGTAGTCCATGCCGTAAACGATGAGCGCTAGGTGTTGGGGACTTTCCGGTCCTCAGTGCCGCAGCAAACGCATTAAGCGCTCCGCCTGGGGAGTACGACCGCAAGGTTGAAACTCAAAGGATTGACGGGGGCCCGCACAAGCGGTGGAGCATGTGGTTTAATTCGAAGCAACGCGAAGACCTTACCAGGTCTTGACATCCTGTGCTACACCTAGAGATAGTGGTTCCCTTCGGGGACGCAAAGACAGGTGGTGCATGCTGTCGTCAGCTCGTGTCGTGAGATGTGGTAGTCCGCACGAGCGCACCCTGTCTAGTGGCATCATTAGTGGGCACTCTAAGAGACTGCGTGACACGAGAGTTGGATGACGTCAAGTCATCATGCCTATGACTGGGCTACACTGCTCATGGCAGTCAACGAAGCTAACCGCTAGGTTAGCGGATC

Above sequence was amplified by 16S rRNA primer set (8F 5’-AGAGTTTGATCCTGGCTCAG-3’; 1492R 5’-GGTTACCTTGTTACGACTT-3’) and used to against the NCBI database. The result indicates that TCI904 belong to *Lactobacillus delbrueckii* subsp. *bulgaricus*.
